# Dose comparison between Gafchromic film, XiO, and Monaco treatment planning systems in a novel pelvic phantom that contains a titanium hip prosthesis

**DOI:** 10.1002/acm2.12141

**Published:** 2017-07-25

**Authors:** Nicholas Ade, F.C.P. du Plessis

**Affiliations:** ^1^ Medical Physics Department University of the Free State Bloemfontein South Africa

**Keywords:** dose perturbations, pelvic phantom, photon beams, prosthesis

## Abstract

The presence of metallic prostheses during external beam radiotherapy of malignancies in the pelvic region has the potential to strongly influence the dose distribution to the target and to tissue surrounded by the prostheses. This study systematically investigates the perturbation effects of unilateral titanium prosthesis on 6 and 15 MV photon beam dose distributions using Gafchromic EBT2 film measurements in a novel pelvic phantom made out of a stack of nylon slices. Comparisons were also made between the film data and dose calculations made on XiO and Monaco treatment planning systems. The collapsed cone algorithm was chosen for the XiO and the Monte Carlo algorithm used on Monaco is XVMC. Transmission measurements were taken using a narrow‐beam geometry to determine the mass attenuation coefficient of nylon = 0.0458 cm^2^/g and for a water‐equivalent RW3 phantom, it was 0.0465 cm^2^/g. The perturbation effects of the prosthesis on dose distributions were investigated by measuring and comparing dose maps and profiles. The magnitude of dose perturbations was quantified by calculating dose enhancement and reduction factors using field sizes of 3 × 3, 5 × 5, 10 × 10, and 15 × 15 cm^2^. For the studied beams and field sizes, dose enhancements between 21 and 30% and dose reductions between 15 and 21% were observed at the nylon‐prosthesis interface on the proximal and distal sides of the prosthesis for film measurements. The dose escalation increases with beam energy, and the dose reduction due to attenuation decreases with increasing beam energy when compared to unattenuated beam data. A comparison of film and XiO depth doses for the studied fields gave relative errors between 1.1 and 23.2% at the proximal and distal interfaces of the Ti prosthesis. Also, relative errors < 4.0% were obtained between film and Monaco dose data outside the prosthesis for 6 and 15 MV lateral opposing fields.

## 1 INTRODUCTION

A common treatment modality for prostate cancer is external beam radiation therapy. It is aimed at delivering a lethal radiation dose to malignant tissues so as to provide a high probability of tumor control while sparing or inducing minimal damage to adjacent normal tissues. Therefore, radiotherapy is directed toward keeping normal tissue complications within acceptable limits while attaining a high therapeutic ratio. However, an increasing number of patients requiring megavoltage photon beam radiotherapy for malignancies in the pelvic or hip region have metal implants or prostheses which could shadow the target and influence the dose distribution leading to a dramatic difference in clinical outcome.^1^, ^2^, ^3^, ^4^

Implants vary in size, composition, and geometry, and the choice of an implant material depends on factors such as corrosion, fatigue resistance, and mechanical strength.^5^ Commonly used metals and alloys for implants include stainless steel, Co‐Cr‐Mo, and Ti.^3^, ^5^, ^6^ Carolan et al. pointed out that Co‐Cr‐Mo alloy with a high relative electron density (6.79–6.9) is likely to have a greater impact on dose distribution than steel (6.55–6.61) and Ti (3.72–3.76) which have low electron densities.^3^ Mesbahi and Nejad, however, observed that the attenuation effect of prostheses is density dependent with steel (*ρ* = 8.1 g/cm^3^) showing the greatest impact followed by Co‐Cr‐Mo (*ρ* = 7.8 g/cm^3^) and Ti (*ρ* = 4.54 g/cm^3^) showing the least effect.^6^ The majority of hip prostheses are composed of Co‐Cr alloys as they are considered to have the best combination of corrosion, fatigue resistance, and mechanical strength.^5^ The high Z and high density of metallic prostheses relative to water yield challenges for radiotherapy dose computation when beams pass through these devices because the dose attenuation through a prosthetic device during pelvic irradiation could be significant.^2^, ^5^, ^6^, ^7^, ^8^, ^9^, ^10^ In addition, the drastic changes in electron scattering characteristics near interfaces due to sudden and extreme changes in density are also challenging for most dose calculation algorithms that are not Monte Carlo (MC) or Acuros as MC and Acuros algorithms can account for the effects of heterogeneities in patient dose calculation.

It is known that there is a decrease in tumor control due to reduced target dose from beam attenuation of the prosthesis^3^ or an increase in complication rates due to the local dose perturbations caused by prosthetic implants.^1^ Perturbations of absorbed dose distribution occur as a result of the increased attenuation of the radiation beam by the prosthetic device and the changes in electron scatter or photon interactions (photoelectric effect and pair production) that occur at the bone–metal interface. Even though oblique beams are usually chosen to minimize or avoid the shadowing effect of the prostheses, this cannot always be accomplished.^4^ It could also cause an increased dose to adjacent structures such as the rectum.^3^, ^11^ A survey of 30 institutions conducted by the AAPM TG‐63 indicated that the number of patients with prostheses, which could affect their radiation therapy, was 1–4% of the total number of patients.^1^ The survey also indicated that there was no general agreement on how to manage the treatment for patients having prostheses. Some institutions ignore their presence, while others try to modify the beam orientation to avoid the prostheses even if extra dose is delivered to adjacent critical structures. With an increasing aging population, it is expected that the number of patients with prostheses is likely to increase due to conditions such as osteoarthritis and dysfunctional hip joints which may require hip replacement. It is understood that the scientific understanding and approach of clinical dosimetry for the presence of metallic prostheses during irradiation of pelvic malignancies is still a challenge.^2^, ^9^, ^10^ Also, the dose perturbation due to these prostheses could affect clinical outcome due to its significance and so it cannot be ignored.^7^, ^8^ As a result, it is necessary to expand the information available in literature with current data which is the motivation of the present study.

A number of researchers have attempted to quantify the dose perturbations due to prostheses by performing dose measurements either in phantom (usually liquid or solid water phantoms) containing the prosthesis or by computing with a treatment planning system (TPS) or Monte Carlo (MC) methods.^2^, ^3^, ^4^, ^6^, ^8^, ^9^, ^10^, ^11^, ^12^, ^13^, ^14^ Biggs and Russell used ionization chamber dosimetry systems and a water tank to measure the effects of a hollow femoral head prosthesis on the dose from lateral fields to the pelvis for megavoltage photon beams.^11^ Sibata et al.^5^ measured dose in water with film and an ion chamber to evaluate prosthesis‐induced attenuation for 6 and 18 MV photons. The prosthetic models had varying size and composition of Co‐Cr, Ti, and stainless steel. Some authors used diodes to measure the dose attenuation for a 10 × 10 cm^2^ 6 MV photon field due to the presence of a Co‐Cr‐Mo hip prosthesis in a water tank.^3^ Others used film to measure the dose attenuation along the length of a Ti alloy hip prosthesis for 6 and 15 MV photon beams.^8^ Spezi et al. evaluated the dosimetric characteristics of commonly used prosthetic implant materials using 6 and 18 MV photon beams. The materials were cut into cylinders and measurements were conducted using ion chambers in water and RW3 solid water phantoms. The measured data were compared with calculations based on Monte Carlo treatment planning models. 2 Kung et al. investigated the feasibility of using IMRT for treating patients with metallic prostheses.^12^ Erlanson and Franzén measured the dose distribution effects caused by a hip prosthesis with small silicon diodes for 6, 20, and 50 MV photons when treating pelvic cancer.^14^ Others studied a Ti alloy prosthesis in a water phantom using MC simulations and a TPS calculations to study the perturbations due to metallic implants for 6 and 18 MV photon beams.^4^ As reported by the researchers, the degree of the dose perturbations varied between 2 and 64%. However, the usual use of water phantoms and the lack of much detail are limitations of most the studies. A more convenient approach will be the use of a more realistic tissue‐mimicking medium such as a water‐equivalent solid pelvic phantom for dose perturbation measurements for a meticulous and systematic study.

In this paper, the dose perturbation effect of Ti prosthesis for 6 and 15 MV photon beams was meticulously studied in a novel realistic pelvic phantom consisting of a stack of nylon slices with bone and Ti embedded in each layer to form a unilateral Ti prosthesis. Dose measurements were made with Gafchromic film to determine dose perturbation factors for a range of field sizes. Single‐ and bilateral‐field film studies were also compared with dose calculations using a CMS XiO and Monaco TPSs. To the best of our knowledge, reports for dose perturbation data for a Ti prosthesis evaluated in such a pelvic phantom for 6 and 15 MV photon beams are scarce. Also, a comparison of Monaco TPS against film measurements for a study involving Ti prosthesis has not been reported previously.

## 2 METHODS

### 2.A The pelvic phantom

**Figure 1:**
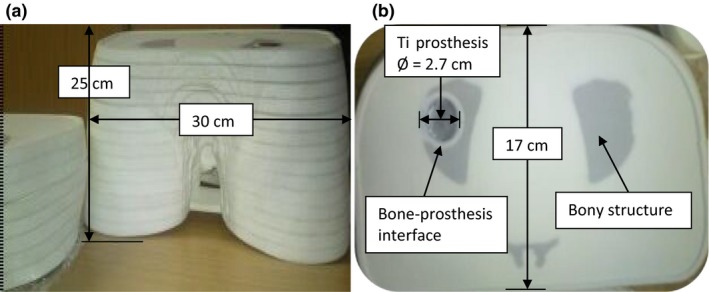
The novel pelvic phantom (a) with unilateral Ti prosthesis on the left (b).

The phantom is locally developed with a built‐in Ti hip prosthesis and was designed for film dosimetry as shown in Fig. 1. It consists of a stack of 25 Nylon‐12 slices of which some are fitted with Ti disks to form a unilateral Ti prosthesis. Nylon‐12 is a polymer and has the formula [(CH_2_)_11_CONH]_n_. The nylon slices are numbered from top to bottom and each is 1 cm thick. The pelvic phantom is 25 cm in height, 30 cm in width, and 17 cm thick, and also contains bony structures including the spinal cord and pelvic bone. The constituents and material compositions (% by mass) of tissue‐equivalent substitutes used for the bony structures include: a base of Araldite GY‐6010 epoxy resin (36.4) with a Jeffamine T‐403 hardener (14.6); and filler materials which comprise silicon dioxide (25.5) and calcium carbonate (23.5).^15^ As the phantom is designed for film dosimetry, removable inter‐slice plastic plate templates were manufactured to allow precise film cutting that could fit between the slices for measurements. The design of the phantom is such that air gaps between the nylon slices which could influence dose measurements are minimized. This is further achieved by using clamps to fasten the nylon slices so as to keep the phantom airtight during measurements. The diameter of the Ti disk in the plane of measurement considered in this study is 2.7 cm [Fig. 1 (b)] and the width of the bone material on the opposite side is about 4.5 cm. There is a thin layer of tissue material (nylon) between the prosthesis and bone, which forms the bone–prosthesis interface.

### 2.A.1 Nylon water‐equivalence

**Figure 2:**
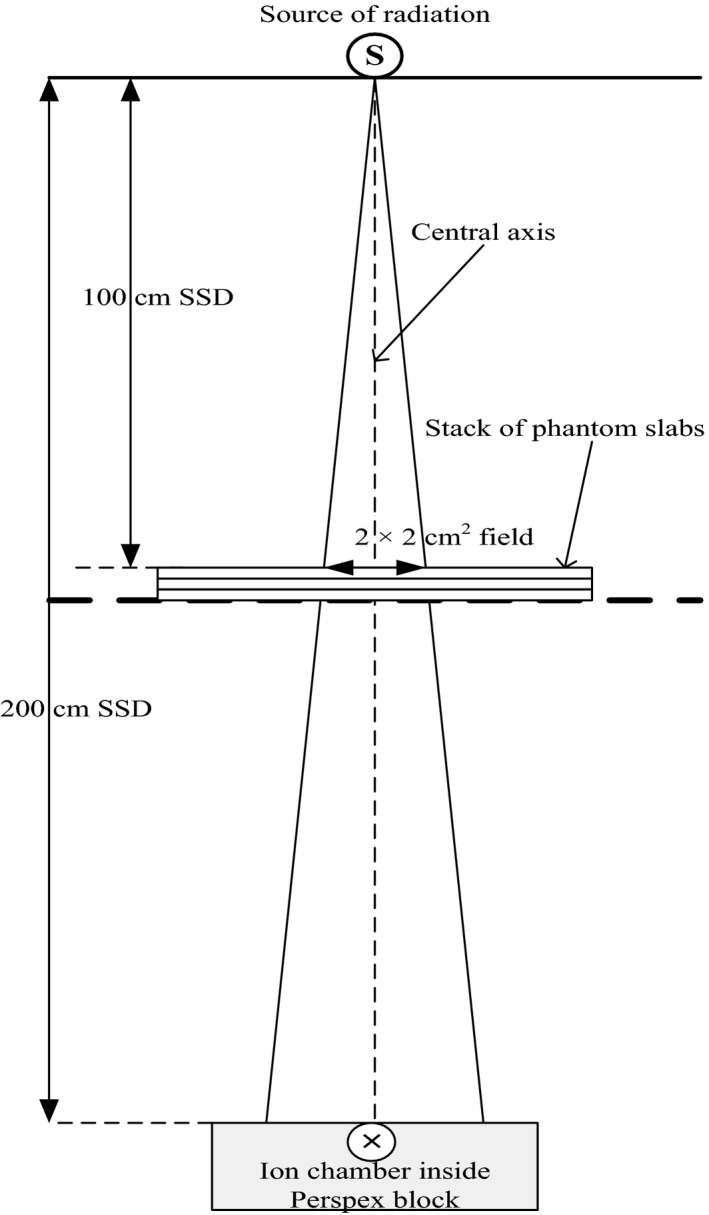
A schematic setup of narrow‐beam geometry for 6 MV transmission measurements

The water‐equivalence of Nylon‐12 was established by measuring central axis (CAX) transmission data through slabs of this material as well as water‐equivalent RW3 slabs.^16^, ^17^ Measurements were made in narrow‐beam geometry using a 6 MV photon beam and a 0.6 cc Farmer‐type ion chamber connected to a PTW UNIDOS E electrometer. The chamber was housed inside a block of Perspex with 0.8 cm buildup. The block, containing the chamber, was placed at an SSD of 200 cm and 6 MV transmission measurements were taken with the phantom slabs placed at an SSD of 100 cm for a set field size of 2 × 2 cm^2^ defined at 100 cm SSD (Fig. 2). 300 monitor units (MU) were set up in each measurement to ensure high signal‐to‐noise ratio. Transmission measurements (*R*_*x*_) were made with different thicknesses of attenuating material for Nylon‐12 and RW3, ranging from 1 to 10 cm in steps of 1 cm.

From these measurements and the application of exponential attenuation law (1), the linear attenuation coefficient (*μ*) was calculated, as well as the mass attenuation coefficient (*μ*_*m*_ *= μ/ρ*).^18^, ^19^ A density value of 1.01 g/cm^3^ was used for Nylon‐12.

(1) $$R_{x}/R_{0}\;=\;\exp\left(-\mu .x\right)=T_{x}$$

In eq. (1), *R*_*x*_ is the ionization signal transmitted through the attenuating phantom material of thickness x (cm), *R*_*o*_ is the initial open beam signal, and *T*_*x*_ is the transmission factor. The linear attenuation coefficient was then determined from a least square fit of an exponential function through the transmission data points on a *T*_*x*_ vs *x* graph.

As electron scatter characteristics are most significant near water and bone–prosthesis interfaces as transient charge particle equilibrium is disturbed at tissue/bone–prosthesis interfaces, another dosimetric parameter to consider for the water‐equivalence of nylon is to evaluate its electron stopping power characteristics relative to water. Table 4 of the AAPM TG‐21 provides a list of ratios of average, restricted stopping powers of medium to air for some materials including nylon for photon spectra ranging from 2 to 45 MV.^20^ The discrepancy between the values of water and nylon are within 1% implying that nylon and water have similar stopping power characteristics.

### 2.B Film calibration and phantom measurement

All dose measurements reported in this study are from exposures of Gafchromic^®^ EBT2 films (manufactured by Ashland Specialty Ingredients) in 6 and 15 MV photon beams produced by an Elekta Precise linac. Film calibrations were performed to convert optical density (OD) to dose. A rational function of the type depicted in eq. (2) was employed:^21^, ^22^

(2) X(D)=a+[b/(D−c)]

Where *X(D)* is the film response at dose *D*, and *a, b*, and *c* fitting parameters obtained from a least square optimization method. Two batches of film were used (Lot #s: 01201501 and 08221302) and from each one, a sheet of film was taken and subdivided into pieces of 10 × 4 cm^2^. The orientation of each film piece was marked. A piece was placed inside a 30 × 30 cm^2^ RW3 slab phantom at 10 cm depth on the CAX of a 10 × 10 cm^2^ 6 MV photon beam at 100 cm SSD. It was then irradiated. The process was repeated 5× and the sequence of MUs set was 0, 75, 150, 300, and 360. This corresponded to dose values between 0 and 244.44 cGy at 10 cm depth in RW3. The film pieces were scanned 24 hr post exposure to allow for polymerization.

Each film piece was placed in the same location on the scanner bed before multiple scans of it was taken in succession to obtain mean values over an invariant region of interest (ROI = 7 × 3 cm^2^) in its center.^23^ This avoided OD measurement artifacts near film edges.^24^ The process was repeated for all film pieces. An Epson Perfection V330 Photo flat‐bed document scanner with a resolution of 72 dots per inch (dpi) was employed to read the films. Film images were scanned as raw 48‐bit RGB (16 bits per color) and saved in tagged‐image‐file format (TIFF) similar to procedures reported in literature.^23^, ^25^, ^26^ These images were processed using information in the 16 bit red (R) channel of the RGB tiff images. The delivered dose D versus measured OD was then fitted employing the analytical function depicted in (2). The OD was determined from the pixel reading in similar procedures as reported elsewhere.^23^

### 2.C Dose distribution EBT2 film measurements

**Figure 3:**
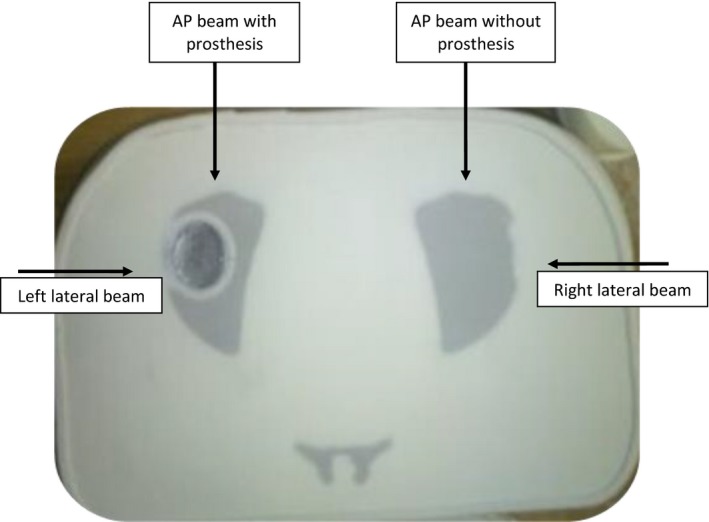
Beam setups/directions for dose perturbation measurements. A 10 × 10 cm^2^ field was used for lateral beams and field sizes of 3 × 3, 5 × 5, 10 × 10, and 15 × 15 cm^2^ were used for AP beams.

The dose perturbation caused by the prosthesis was investigated for 6 and 15 MV photon beams. The phantom was first irradiated with 6 MV opposing fields (left and right lateral) as shown in Fig. 3. The plane between phantom slices 11 and 12 [Fig. 1(b)] was chosen for film measurement. The phantom was set up with the prostate positioned at the isocenter and irradiated to a dose of 300 cGy with the two opposing 10 × 10 cm^2^ fields.

Quantification of the prosthesis‐induced dose perturbation was expressed through dose perturbation factors (DPFs) which is defined as the ratio of the doses with and without the presence of the prosthesis. To measure this, the pelvic phantom was placed in the supine position, and 6 and 15 MV depth dose distributions were acquired using single AP beams for field sizes of 3 × 3, 5 × 5, 10 × 10, and 15 × 15 cm^2^ at an SSD = 91.5 cm. Film measurements were acquired for these AP beams using the symmetrical property of the phantom that has the same effect as measurements with the prosthesis (left side) and without the prosthesis (right side) as indicated in Fig. 3. The MUs chosen for each beam was such that 300 cGy was delivered at 100 cm SAD, located 8.5 cm below the phantom surface for each film measurement. The measurement orientation was along the axial slices of the phantom. The dose distributions of 6 and 15 MV for bilateral beam arrangements that comprise a parallel opposed pair of equal weights were also measured. For each beam arrangement, EBT2 film was inserted in the measurement plane of the pelvic phantom. A single fraction dose of 5 Gy was delivered at isocenter. Beam angles of 270° and 90° were used for the beam arrangements which employed left and right lateral fields.

### 2.D Treatment planning dose calculation

The above film dose measurements were compared against counterpart dose calculations produced by a CMS XiO (v. 4.62) treatment planning system (TPS) for 6 and 15 MV photon beams for the single AP fields of 3 × 3, 5 × 5, 10 × 10, and 15 × 15 cm^2^. Prior to this, the phantom was CT‐scanned with a Toshiba Aquilion^TM^ 16LB CT scanner after which the DICOM images were imported into the TPS. A superposition algorithm was used for dose calculation employing a grid size of 2.0 mm.

Dose distributions for the two 6 and 15 MV parallel opposed pairs described in section II C were also calculated. In this case, an Elekta Monaco TPS utilizing the X‐ray voxel Monte Carlo (XVMC) (v. 5.00.00) algorithm was used for dose calculations. The treatment plans were generated for the pelvic phantom with inserted film and delivered on the linac. The field size was set to conform to a target contoured on the DICOM images of the phantom. The Monaco TPS calculated dose distributions were then compared to those measured with film in the pelvic phantom that contains the prosthesis. The relative errors (*δ*) between film measurements and TPS calculations were computed as follows.

(3) $$\delta =[\left(D_{Film}-D_{TPS}\right)/D_{Film}]\times 100$$

## 3 RESULTS

### 3.A Transmission measurements

**Figure 4:**
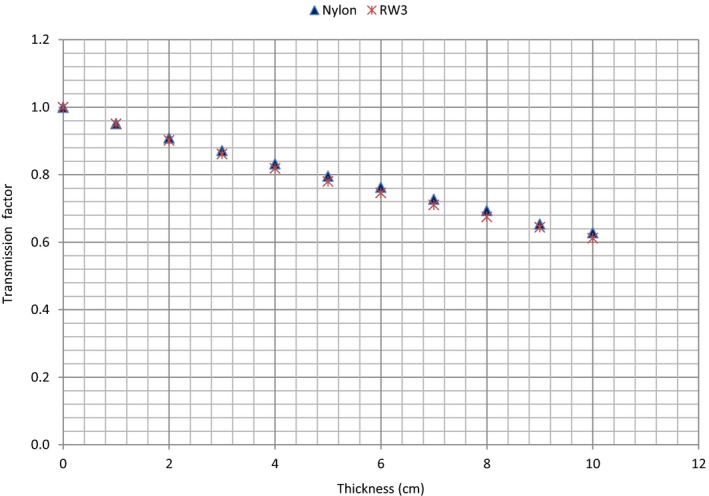
Transmission factors as a function of thickness for Nylon‐12 and RW3 slabs.

Transmission factors for different thicknesses of nylon and water‐equivalent RW3 materials are shown in Fig. 4. Table 1 shows the calculated values of the linear attenuation and mass attenuation coefficients (*μ* and *μ*_*m*_ *)* for the respective density values of 1.045 g/cm^3^ 16, 18 and 1.01 g/cm^3^ for RW and Nylon‐12. A difference of 1.5% was found between the measured *μ*_*m*_ values of Nylon‐12 (*μ*_*m*_ = 0.0458 cm^2^/g) and RW3 (*μ*_*m*_ = 0.0465 cm^2^/g). In literature, *μ*_*m*_ values of 0.0470 and 0.04767 cm^2^/g for RW have been reported for a 6 MV photon beam with the former value determined in a 5 × 5 cm^2^ field.^16^, ^17^ The reason for the small discrepancy (≈1.1%) between the *μ*_*m*_ values of RW obtained in this study and that reported in literature (*μ*_*m*_ = 0.0470 cm^2^/g) could be due to the different field sizes used. In this study, a 2 × 2 cm^2^ field was used to establish narrow‐beam geometry (and the uncertainty in the data [the standard deviation of the average values of three measurements] was <1%), while a 5 × 5 cm^2^ field was reported in literature. Also, *μ* values of 0.0491 and 0.0498 cm^−1^ have been reported for RW3 compared to the value of 0.0486 cm^−1^ obtained in the present study.^16^, ^27^

**Table 1:** Linear and mass attenuation coefficients of Nylon‐12 and water‐equivalent RW3 plastic materials determined using a 6 MV photon beam

Phantom material	*μ* (cm^−1^)	*μ*_m_ (=(*μ*/*ρ*)) (cm^2^/g)
Nylon 12	0.0463	0.0458
RW3	0.0486	0.0465

### 3.B Film calibration

**Figure 5:**
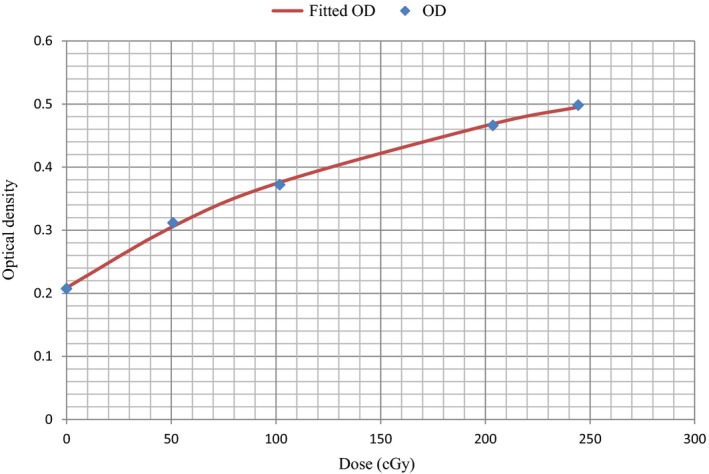
Calibration curve for Gafchromic EBT2 film using the red channel tiff image pixel data

Calibration curves for the red channel tiff image pixel data are shown in Fig. 5 with points corresponding to the measured mean OD at the corresponding dose. Superimposed on these points is the fitted rational function (eq. (2)) with fitting parameters of *a* = 0.794, *b* = −149.260, and *c* = −255.068.

### 3.C Dose distribution measurements and quantification of dose perturbations

**Figure 6:**
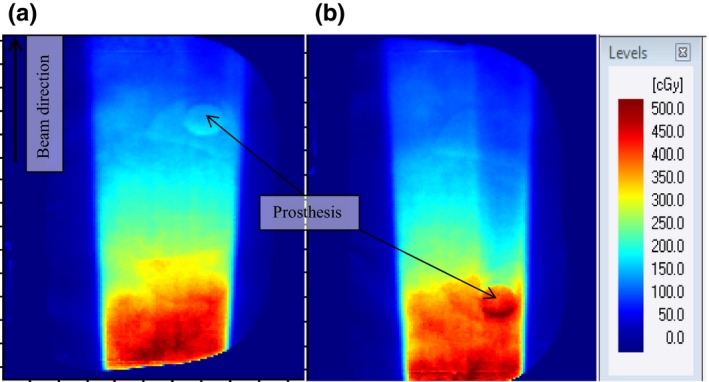
Dose maps taken with the single right (a) and left (b) lateral beams for a 10 × 10 cm^2^ field illustrating the effect of prosthesis on 6 MV photon beam dose distributions.

Figures 6(a) and 6(b) show 6 MV dose maps for the single right and left lateral beams respectively measured for a 10 × 10 cm^2^ field. The beam direction for the two datasets is indicated on Fig. 6(a). The color palette shows the intensity or dose variation on the dose images. For each map, the dose increases at the interface between Ti and Nylon‐12 on the proximal side and decreases in the distal region of the prosthesis. This is due to electrons backscattering from the interface into the incoming beam direction and attenuation of the photon beam in the prosthesis respectively.^1^, ^3^, ^7^, ^8^, ^11^, ^28^

**Figure 7:**
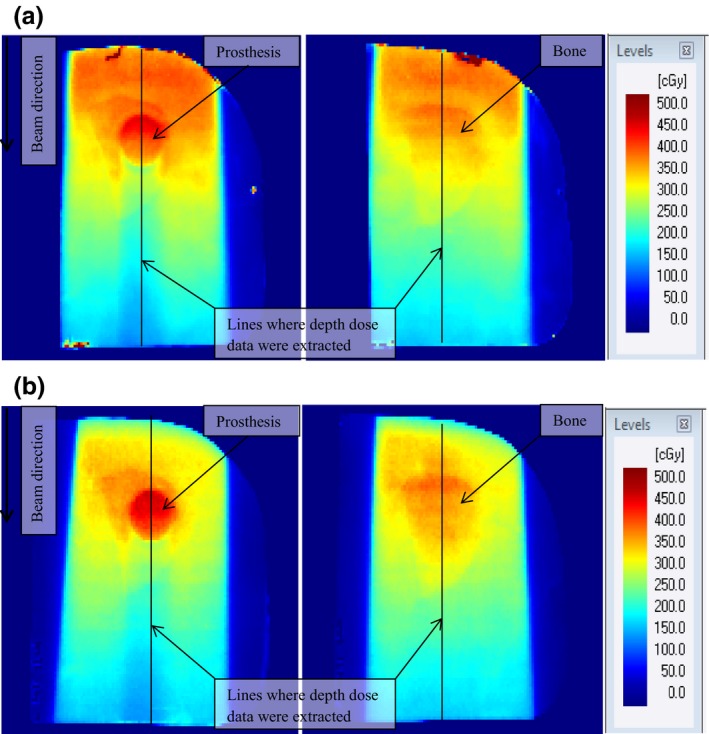
Dose maps taken with (left column) and without (right column) prosthesis obtained using AP beams for a 10 × 10 cm^2^ field, illustrating the effect of prosthesis on (a) 6 MV and (b) 15 MV photon beams.

Figures 7(a) and 7(b) show 6 and 15 MV photon beam dose maps, respectively for the AP beams, with and without the prosthesis for a field size of 10 × 10 cm^2^. Similar to the observation made in Fig. 6, the dose increases at the nylon–prosthesis interface on the proximal side and decreases in the distal region of the prosthesis.

**Figure 8:**
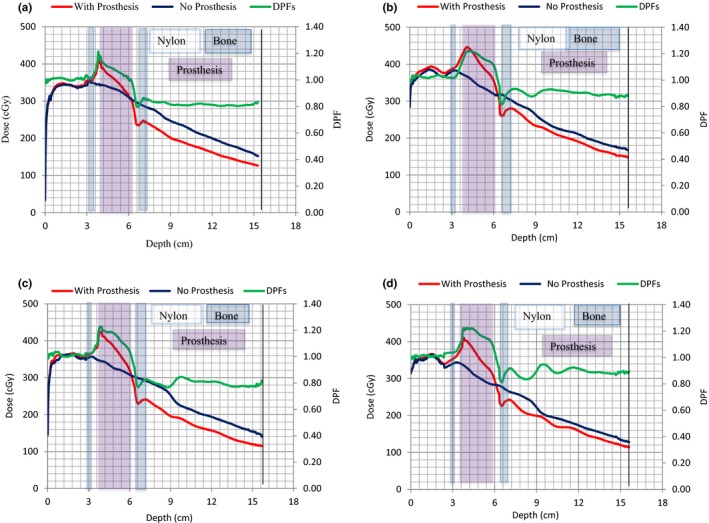
(a)–(d). Variations of 6 MV beam depth dose data with and without prosthesis. Dose perturbation factors with depth are indicated by the green line. Field sizes of 15 × 15, 10 × 10, 5 × 5, and 3 × 3 cm^2^ were studied in Figs. 8(a)–8(d), respectively.

**Figure 9:**
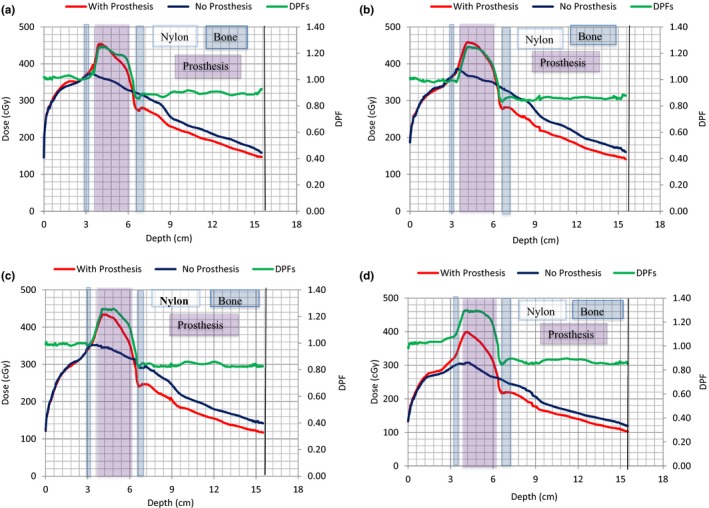
(a)–(d). Variations of 15 MV photon beam depth dose data with and without prosthesis. Dose perturbation factors with depth are indicated by the green line. Field sizes of 15 × 15, 10 × 10, 5 × 5, and 3 × 3 cm^2^ were studied in Figs. 9(a)–9(d), respectively.

Comparison of the 6 and 15 MV photon beam dose maps based on the intensity or color levels reveals that the prosthesis is receiving a higher dose when irradiated with the 15 MV than with the 6 MV beam. The dose increase and beam attenuation depicted by the dose images for the 10 × 10 cm^2^ 6 and 15 MV photon fields were also observed for the 3 × 3, 5 × 5, and 15 × 15 cm^2^ fields. In Figs. 7(a) and 7(b) are also lines along the beam central axis that indicate the depth dose data sampling points used in Figs. 8(a)–8(d) and 9(a)–9(d).

Figures 8(a)–8(d) and 9(a)–9(d) show depth dose data for the 6 and 15 MV photon beams for the field sizes indicated, respectively. Regions consisting of Nylon‐12, bone, and Ti are also indicated. The effects of the prosthesis on the dose distributions are clearly visible as its presence causes significant dose alteration (red lines) compared to the cases with no prosthesis (blue lines). There is dose enhancement at the proximal side of the prosthesis and dose reduction in the distal region relative to the bony structure alone. The dose enhancement depicted by the peak at the proximal side is due to electron backscatter at the tissue–prosthesis interface, while the dose reduction on the distal side of the prosthesis is due to attenuation of the primary beam passing through it.^1^, ^3^, ^7^, ^8^, ^11^, ^28^

Figures 8 and 9 also show that there is a dip in dose immediately after the prosthesis in the distal region that is followed by a dose escalation and then the standard dose fall off is seen. These observations are due to loss of electron fluence immediately after the prosthesis as most of the electrons generated in the prosthesis stay within it, and at the same time, the nylon provides very little backscatter.

DPFs were calculated from the depth doses sampled in Figs. 7(a) and 7(b) data and are shown by the green curves in Figs. 8(a) and 9(d). A DPF = 1.0 indicates the borderline between dose enhancement (DPF > 1.0) and dose reduction (DPF < 1.0). From these figures, the DPFs > 1.0 occur inside the prosthesis and on its proximal side, while DPFs < 1.0 occur in the distal region of the prosthesis. In Table 2, maximum DPFs ranged between 1.21 and 1.23 corresponding to dose enhancements of 21% and 23% for 6 MV, and between 1.25 and 1.30 (dose enhancements of 25 and 30%) for 15 MV beam, respectively, for field sizes of 15 × 15 and 3 × 3 cm^2^. Similarly, minimum DPFs ranged between 0.79 and 0.82 (21 and 18% dose reductions) for 6 MV beam. For 15 MV, it ranged between 0.82 and 0.85 (dose reductions of 18 and 15%), occurring for field sizes of 5 × 5 and 3 × 3 cm^2^, respectively. The uncertainty in these data is 2% (the standard deviation of the average values of three measurements).

**Table 2:** Average values of maximum and minimum dose perturbation factors. The uncertainty (standard deviation) in the data is 2%

Field size (cm^2^)	6 MV photon beam		15 MV photon beam	
	Max. DPF	Min. DPF	Max. DPF	Min.
15 × 15	1.21	0.81	1.25	0.84
10 × 10	1.22	0.81	1.25	0.83
5 × 5	1.22	0.79	1.26	0.82
3 × 3	1.23	0.82	1.30	0.85

The DPF values for the four fields are shown in Table 2. It was also observed that the maximum values of the DPFs in the phantom (excluding the prosthesis) occurred at the beam entrance region of the Ti prosthesis. The prosthesis itself receives higher dose from 15 MV beams compared to 6 MV beams. This observation could be attributed to the longer range of more energetic electrons generated in the beam proximal to the prosthesis in the 15 MV beam. This actually means that the Ti prosthesis intercepts a higher proportion of the secondary electron fluence, and thus more dose is deposited in the 15 MV than for 6 MV.

**Figure 10:**
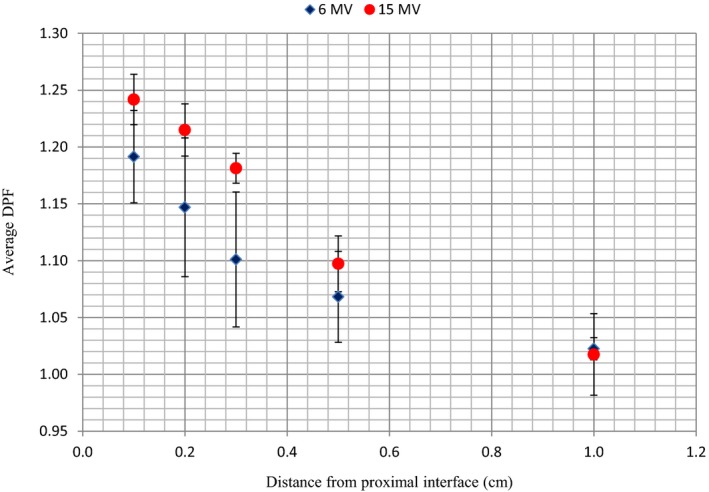
Variation of average values of dose perturbation factor (DPF) with distance from the nylon–prosthesis proximal interface for 6 and 15 MV beams. The error bars show the uncertainty (standard deviation of the mean values) in the data.

Table 3 shows DPF values in the proximal region of the Ti prosthesis in relation with distance from its interface. From Table 3, the following observations can be made: (a) regardless of field size and energy, the DPF decreases with distance from the proximal surface of the prosthesis. For instance, for the 5 × 5 cm^2^ 15 MV field, the DPF varies from 1.22 at 0.1 cm to 1.00 at 1 cm from the interface, (b) the variability in DPF with field size also tends to be lesser further away from the surface regardless of energy, and (c) at any given distance from the prosthesis, the sensitivity of the DPF to field size variation is found to be greater for lower energies. The narrow distance window through which the proximal DPF is observed suggests a low range of backscatter electrons which in turn suggests low‐energy backscatter electrons.^28^ The range of backscattered electrons is also dependent on the photon energy. Figure 10 shows the average values of the DPF for all four‐field sizes as a function of distance from the proximal interface. It is observed that the DPF due to backscatter is higher for 15 MV compared to 6 MV.

**Table 3:** Variation of the DPF with distance from the tissue–prosthesis interface on the proximal side of the prosthesis. The uncertainty in the data is 2%

Distance (cm)	Field size (cm^2^) for 6 MV				Field size (cm^2^) for 15 MV			
	3 × 3	5 × 5	10 × 10	15 × 15	3 × 3	5 × 5	10 × 10	15 × 15
0.1	1.20	1.22	1.21	1.12	1.27	1.22	1.24	1.24
0.2	1.12	1.19	1.20	1.07	1.23	1.19	1.20	1.23
0.3	1.09	1.10	1.17	1.05	1.20	1.17	1.18	1.18
0.5	1.05	1.07	1.11	1.02	1.13	1.09	1.10	1.07
1.0	1.03	1.02	1.01	1.01	1.07	1.00	0.99	1.01

### 3.D Dose distribution measurements *vs* TPS dose calculations

**Figure 11:**
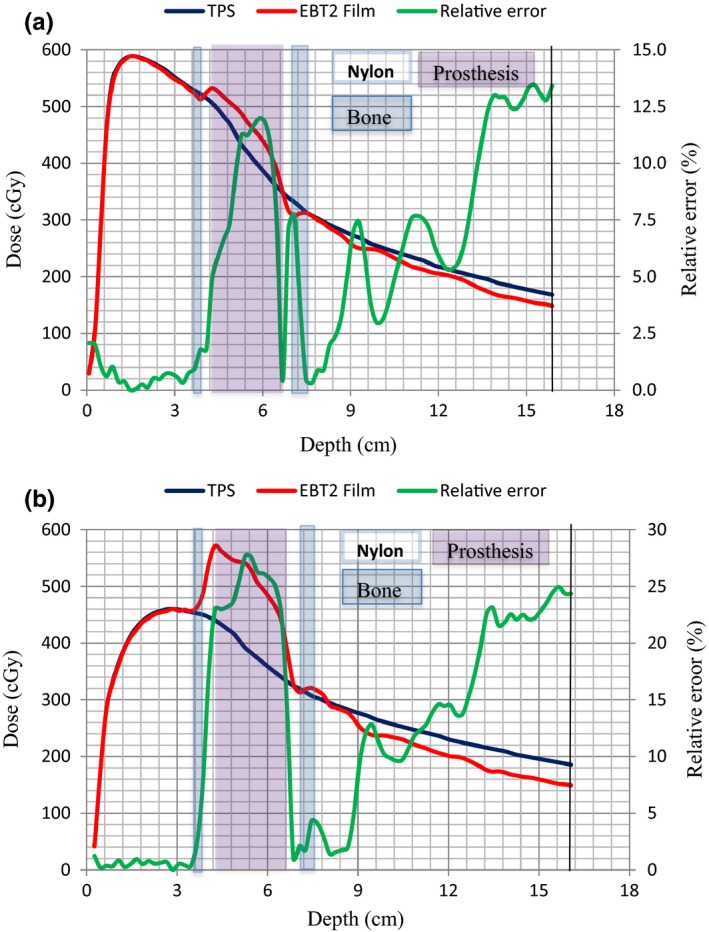
(a) and (b). Comparisons of photon beam dose distributions determined by film measurements and XiO TPS calculations for a field size of 5 × 5 cm^2^. Data are shown for 6 MV (a) and 15 MV (b) beams.

Dose measurements for single AP fields were compared against dose calculations from a CMS XiO TPS as shown in Figs. 11(a) and 11(b) for 6 and 15 MV photon beams, respectively. Depth dose curves produced by the TPS and Gafchromic film measurements are presented for a 5 × 5 cm^2^ field size. The depth dose data are normalized at the depth of dose maximum (d_max_) and regions consisting of nylon, bone, and prosthesis are also indicated.

A comparison of the TPS calculated depth dose curves (blue) and those measured with Gafchromic film (red) show that the TPS data do not match the measured dose over the full‐depth range from the proximal interface of the Ti prosthesis. The largest deviations occur where the measured dose is higher inside the Ti prosthesis. If the measured data are taken as the gold standard, then the TPS superposition algorithm underestimates the dose in the prosthesis and does not account efficiently for attenuation distally to the prosthesis for the 5 × 5 cm^2^ field at 6 and 15 MV. Similar observations as above were made for the 3 × 3, 10 × 10, and 15 × 15 cm^2^ field sizes and presented in Table 4. The relative errors between the film and XiO TPS dose data calculated at the proximal and distal interfaces of the Ti Prosthesis are presented in Table 4. For the studied fields and beams, relative errors between 1.9 and 23.2% were obtained at the proximal interface with higher values occurring at 15 MV compared to the 6 MV beam. Likewise, relative errors between 1.1 and 14.7% were obtained at the distal interface with higher values occurring at 6 MV compared to the 15 MV beam. The results indicate that the TPS is unable to account for the dose perturbations (due to changes in scatter characteristics) near the proximal and distal interfaces. It was inaccurate for both energies with values reaching 14.7% at the distal interface for 3 × 3 cm^2^ 6 MV field and 23.2% at the proximal interface for 5 × 5 cm^2^ 15 MV field.

**Table 4:** Relative errors (%) for depth dose values between film measurements and XiO TPS determined at the proximal and distal interfaces of the prosthesis

Field size (cm^2^)	6 MV photon beam		15 MV photon beam	
	Proximal	Distal	Proximal	Distal
15 × 15	4.1	5.1	15.2	1.5
10 × 10	7.3	7.7	19.3	1.1
5 × 5	4.9	7.7	23.2	2.1
3 × 3	1.9	14.7	21.2	3.8

**Figure 12:**
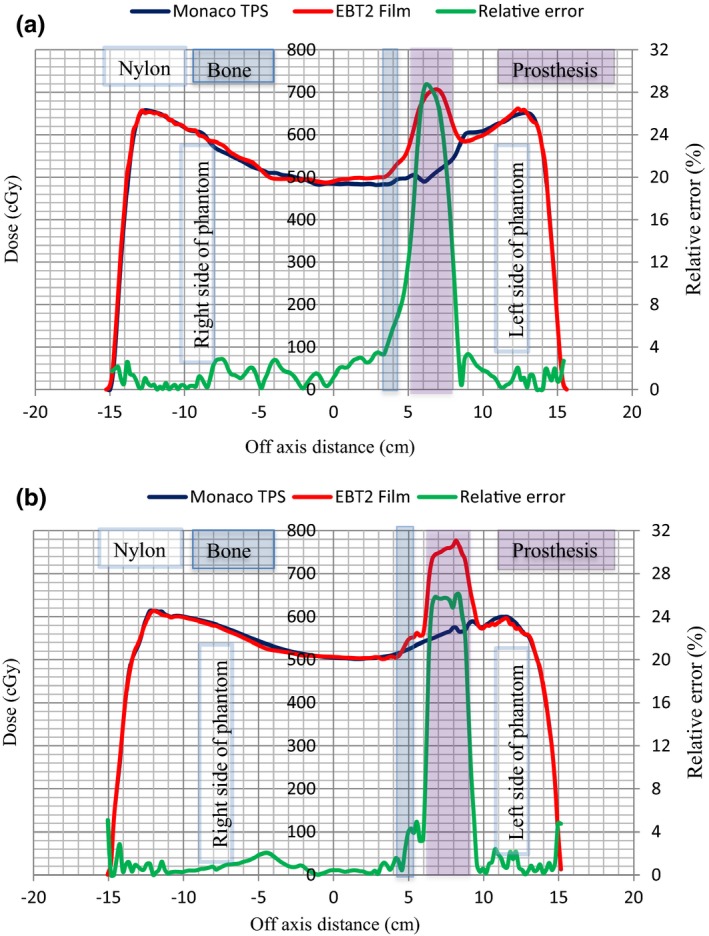
(a) and (b). Comparisons of dose profiles determined by film measurements and Monaco TPS calculations for two opposing lateral fields. Data are shown for 6 MV (a) and 15 MV (b) photon beams. The left and right sides of the pelvic phantom are labeled on the graphs.

The treatment of the prostate may involve the use of lateral fields when 3D conformal planning is considered. Figures 12(a) and 12(b) show 6 and 15 MV dose profiles, respectively, for lateral opposed fields obtained through EBT2 film measurements and dose calculations based on a Monaco TPS which uses the Monte Carlo (XVMC) algorithm. Superimposed on the profiles are regions that comprised the prosthesis, nylon, and bone (the bone is shown only on the left side of the phantom on the distal side of the prosthesis with respect to the left lateral beam as reflected in Fig. 3). The curves for relative errors between film and Monaco datasets are also plotted on the figures. Relative errors of about 29 and 26% between the film and Monaco dose data were obtained inside the prosthesis for 6 and 15 MV, respectively. The dose increase inside the Ti prosthesis which is not fully accounted for by Monaco TPS is due to incorrect density assignment by the XVMC algorithm. It is known that the XVMC algorithm assigns material properties by mass density for densities in the range 1.0–3.0 g/cm^3^.^29^ Also, XVMC only distinguishes bone‐like and soft‐tissue‐like materials. Above the density value of 3.0 g/cm^3^, assignment of material properties, especially Compton cross sections, and dose computation in the material are incorrect. This could explain the large difference in the dose values observed between the film measurements and those calculated by Monaco inside the prosthesis as the mass density of Ti is 4.54 g/cm^3^. However, of clinical significance is the dose distribution outside the prosthesis. As shown on these figures, relative errors of <4.0% were obtained between the film and Monaco dose data for both 6 and 15 MV beams at all other locations in the phantom.

## 4 DISCUSSION

The limitations of various TPS algorithms at interfaces with different atomic numbers near the hip prostheses have been observed.^4^, ^10^ Comparing dose distributions in the vicinity of various hip prostheses calculated by Monte Carlo (MC), superposition, and pencil beam algorithms, Keall et al. pointed out that superposition and pencil beam algorithms are expected to predict a higher dose than MC outside the prosthesis.^10^ Using a commercial 3D TPS (CADPLAN 6.27) in comparison with MC simulations, Ding and Yu reported that CADPLAN (based on equivalent path length method, EPL) is inaccurate in calculating doses for beams passing through high‐density prostheses.^30^ The CADPLAN underestimated the attenuation of hip prostheses (Ti or steel) due to its limitation in assigning the electron density of the prostheses which led to an overestimation of the target dose by 14 and 5% for a typical four‐field box and an eight‐field technique, respectively. Evaluating the accuracy of Alfard TPS in comparison with MC simulations for a 9 MV photon beam, Mesbahi and Nejad reported that Alfard TPS calculations can cause 3, 21, and 26% over‐dosage for points beyond 5 cm depth for Ti, Co‐Cr‐Mo, and steel prostheses, respectively, with the error increasing with depth.^6^ The data presented in this study highlight that treatments where beams pass through the prosthesis should be avoided whenever possible or appropriate corrections for the influence of the prosthesis should be included in dose calculations using the TPS algorithm.

Dose perturbations due to the presence of high Z materials in homogenous media are well documented in literature mostly for 6 and 18 MV photon beams (Table 5), but with considerable variation from one study to another. The magnitude of the perturbations varies between 2 and 64% depending on the size, thickness, mass density, design, and composition of the prosthesis as well as the differences in multiple scatter of the secondary electrons and the incident beam energy.^1^, ^2^, ^4^, ^8^, ^9^, ^10^, ^11^, ^12^, ^13^, ^14^ Additionally, most data are limited to the perturbation effect due to photon attenuation behind an inhomogeneity usually placed in normal water or plastic phantoms. In this study, a novel pelvic phantom that simulates patient geometry with a built‐in Ti prosthesis is employed. Dose perturbations were systematically investigated along depth dose curves and not just at the beam entry or beam exit of the prosthesis as often reported in literature. Presented in Table 5 are some of the documented values of dose perturbations for various prostheses and photon energies. For a Ti alloy, a dose increase of 25% at 50 MV and dose reductions between 10 and 40% at 20 MV beams have been reported at various depths between 8 and 10 cm by Erlanson and Franzén.^14^ Using a scanning film dosimeter, Eng reported dose attenuations at different thicknesses along the length of a Ti alloy prosthesis which ranged from 32 to 60% for 15 MV and from 39 to 64% for 6 MV photon beams.^8^ Also, for a Ti alloy, maximum attenuations which ranged 0.26–0.28 and 0.17–0.20 at a depth of 10 cm were reported by Sibata et al. for 15 × 15 cm^2^ 6 MV and 18 MV photon beams, respectively.^5^

**Table 5:** Dose perturbations reported in literature for various prostheses and photon energies. References are indicated as superscripts and the depths (in cm) where the perturbations were obtained are indicted in brackets

Material	Max. dose enhancement (%)			Max attenuation or reduction (%)		
	6 MV	15 MV	18 MV	6 MV	15 MV	18 MV
Ti	–	–	–	26–285 (10)	–	17–205 (10)
Ti	–	–	–	39–648	32–608	–
Co‐Cr‐Mo	353 (15)	–	–	343 (15), 505 (10)	–	375 (10)
Stainless Steel	–	–	–	445 (10)	–	415 (10)
Platinum	42–5128 (8)	–	–	–	–	–
Lead	341	–	451	–	–	–

In this study, the following observations can be made from Table 2: (a) the max DPF, which reflects the maximum dose enhancement at the proximal side of the prosthesis, was found to be higher for 15 MV and larger for smaller fields, (b) the min DPF, which is as a result of the most attenuation on the distal side of the prosthesis was found to be so for 6 MV. The min DPF did show some field size dependence although the trend was a decrease with decrease in field size and then an increase again, (c) the dose enhancement on the proximal side was found to be higher for 15 MV as opposed to 6 MV because of the longer range of the more energetic electron backscatter generated in the beam proximal to the prosthesis in the 15 MV beam, and (d) the dose decrease on the distal side was found to be more for 6 MV as opposed to 15 MV due to more attenuation of primary photons for lower energies.

The findings of this study thus agree with other studies where it is found that the dose attenuation decreases with increase in photon energy (Table 5).^5^, ^8^ Also, the min DPF reported in this study for both 6 and 15 MV beams shows a dip at 5 × 5 cm^2^ field and then increases again. A similar effect has been observed by Rustgi et al. who reported that for an aluminum interface in small 6 MV photon fields used in stereotactic radiosurgery, the dose reduction factors initially decreased with increasing field size and then remained constant for fields >25 mm in diameter.^31^ Similar to the present study, the dose enhancement or backscatter dose factor (BSDF) has been reported to increase with photon energy.^1^, ^14^, ^28^ For a platinum implant at the center of a 160 mm diameter water phantom, Cheung et al. reported dose enhancements from 32 to 46%, 42 to 51%, and 60 to 68% for 4, 6, and 10 MV stereotactic beams, respectively.^32^ For a slab inhomogeneity made of lead, the BSDF was observed to increase from 1.34 to 1.45 for photon energies of 6 and 18 MV, respectively.^1^

It has, however, been shown that for lower Z materials such as bone and aluminum, the BSDF is roughly constant with photon energy up to 10 MV and then falls off at higher energies.^28^ For higher Z materials such as lead, the BSDF increased from ^60^Co and peaked at 10 MV.^28^ Little variation of the dose enhancement with field size has been reported.^11^, ^28^ For various materials in photon beams from 6 to 24 MV, the BSDF was found to be constant with field size between 4 × 4 and 20 × 20 cm^2^, except for lead where the BSDF was lower at smaller fields and saturates at 8 × 8 cm^2^.^28^ This field size independence of the BSDF was attributed to electron transport rather than photon backscattering.^28^ The findings of the present study (Table 2), however, show that the dose enhancement at the proximal interface of the prosthesis increases with decrease in field size for both 6 and 15 MV beams. Cheung et al. observed that dose enhancement was also sensitive to both the beam size and beam energy, where larger collimators resulted in smaller dose enhancements because the increase of scattering partially washed out the dose enhancements.^32^ Examination of Table 3 also indicates that there is a field size dependence of the DPF in the proximal region which depends on the photon energy and the distance from the proximal interface of the prosthesis.

## 5 CONCLUSION

In this study, the dose perturbation effects of titanium prosthesis on 6 and 15 MV photon beam dose distributions were investigated using Gafchromic EBT2 film measurements in a novel pelvic phantom (made out of nylon slices) for a range of field sizes. Through transmission measurements, the pelvic phantom was shown to be water‐equivalent. The data presented indicate that dose increases between 21 and 30% occurred at the interface between the prosthesis and tissue on the proximal side of the prosthesis. Also, dose reductions between 15 and 23% were observed in the distal region or shadow of the prosthesis. The magnitude of the dose perturbations was observed to vary with beam energy and was found to show a small variation with field size. DPFs were also observed to fall off with distance from the proximal interface and approached unity as the phantom surface was approached. A comparison of TPS (CMS XiO and Elekta Monaco) calculated dose data and those measured with Gafchromic film suggested that both XiO and Monaco TPSs could not accurately predict the effect of the Ti prosthesis. However, Monaco TPS could calculate dose outside the prosthesis to an accuracy of <4% for 6 and 15 MV parallel opposed fields.

## CONFLICT OF INTEREST

The authors declare no conflict of interest.
